# Detection of Venous Thromboembolism by Proteomic Serum Biomarkers

**DOI:** 10.1371/journal.pone.0000544

**Published:** 2007-06-20

**Authors:** Santhi K. Ganesh, Yugal Sharma, Judith Dayhoff, Henry M. Fales, Jennifer Van Eyk, Thomas S. Kickler, Eric M. Billings, Elizabeth G. Nabel

**Affiliations:** 1 National Heart, Lung and Blood Institute, Bethesda, Maryland, United States of America; 2 National Human Genome Research Institute, Bethesda, Maryland, United States of America; 3 Johns Hopkins University School of Medicine, Baltimore, Maryland, United States of America; Academic Medical Center, Netherlands

## Abstract

**Background:**

Available blood assays for venous thromboembolism (VTE) suffer from diminished specificity. Compared with single marker tests, such as D-dimer, a multi-marker strategy may improve diagnostic ability. We used direct mass spectrometry (MS) analysis of serum from patients with VTE to determine whether protein expression profiles would predict diagnosis.

**Methods and Results:**

We developed a direct MS and computational approach to the proteomic analysis of serum. Using this new method, we analyzed serum from inpatients undergoing radiographic evaluation for VTE. In a balanced cohort of 76 patients, a neural network-based prediction model was built using a training subset of the cohort to first identify proteomic patterns of VTE. The proteomic patterns were then validated in a separate group of patients within the cohort. The model yielded a sensitivity of 68% and specificity of 89%, which exceeded the specificity of D-dimer assay tested by latex agglutination, ELISA, and immunoturbimetric methods (sensitivity/specificity of 63.2%/60.5%, 97.4%/21.1%, 97.4%/15.8%, respectively). We validated differences in protein expression between patients with and without VTE using more traditional gel-based analysis of the same serum samples.

**Conclusion:**

Protein expression analysis of serum using direct MS demonstrates potential diagnostic utility for VTE. This pilot study is the first such direct MS study to be applied to a cardiovascular disease. Differences in protein expression were identified and subsequently validated in a separate group of patients. The findings in this initial cohort can be evaluated in other independent cohorts, including patients with inflammatory conditions and chronic (but not acute) VTE, for the diagnosis of VTE.

## Introduction

Venous thromboembolism (VTE) occurs for the first time in ∼100 per 100,000 persons each year in the United States. Approximately one third of patients with symptomatic VTE manifest pulmonary embolism (PE) and two thirds present with deep vein thrombosis (DVT)[1]. Death occurs within the first month in approximately 12% of PE cases and 6% of DVT cases[1]. Current noninvasive testing for VTE includes blood assays of D-dimer. D-Dimer is a fibrin-specific degradation product that signifies endogenous fibrinolysis of cross-linked fibrin. Several studies have demonstrated good negative predictive value of D-dimer assays but poor specificity as a marker of VTE[2], [3]. A more accurate biomarker would be of clinical value since management decisions must be made promptly and accurately[4]–[6].

Multivariate strategies to disease diagnosis have been proposed for risk assessment and clinical decision-making, in diseases such as acute coronary syndromes[7], [8]. The use of multiple markers may provide a more accurate assessment of underlying conditions, compared to single protein markers, especially in the case of hemostasis[9]. We therefore hypothesized that a proteomic biomarker panel consisting of multiple serum proteins assessed simultaneously would enhance the diagnostic accuracy for VTE compared to D-dimer.

Mass spectrometry (MS) is a powerful analytic tool, capable of analyzing complex protein mixtures. Since matrix-assisted laser desorption/ionization time-of-flight (MALDI-TOF) MS can detect a wide range of full length proteins, we hypothesized that a MALDI-TOF MS approach could assay multiple protein markers for the diagnosis of VTE. Protein expression patterns apparent in mass spectra may be useful in a clinical setting if they carry sufficient power as a biomarker in themselves, regardless of whether protein identities are known[10], [11]. In this approach, multiple proteins can be assayed and used in a combinatorial fashion to assess underlying diseases states. Validation of specific protein expression patterns, defined as changes in either protein concentration or post-translational modifications of proteins, can then be carried out in subsequent experiments. However, the appropriate application of MS for this purpose requires a detailed understanding of the sources of variance in mass spectrometric data and use of advanced computational methods[12]. We systematically characterize sources of variation in the application of direct MS to the analysis of whole serum and address computational issues using a stringent approach to the analytic methods developed for this study, guided by a series of simulation experiments (Billings, E, unpublished data, 2006).

To test our hypothesis that protein expression patterns measured across the serum proteome would have potential diagnostic utility in VTE, we developed and applied a protein marker assessment that uses MALDI-TOF MS and new computational approaches in a cohort of hospitalized patients undergoing radiographic evaluation for VTE. Diagnostic accuracy of D-dimer assays has been shown to be especially problematic in this population[13]. Comparing the MS results to other currently available VTE biomarkers in the same cohort of patients demonstrates enhanced diagnostic precision of serum protein markers identified by direct MS. Further, to address whether direct MS methods detect changes in protein expression, as opposed to noise or other chemical signatures, we validated differential protein expression among patients with VTE by performing 2-dimensional gel electrophoresis (2DGE) in a subset of the cohort, demonstrating that differences in serum protein expression are present in patients with VTE.

## Materials and Methods

### Study Subjects

We studied 116 inpatients at Johns Hopkins Hospital undergoing radiographic evaluation for VTE. Serum samples were obtained from unselected inpatients at the time of radiographic testing under a protocol which evaluated the diagnostic value of clinically approved blood assays to detect VTE[13]. The protocol was approved by the hospital institutional review board, and informed consent was obtained from all participants. Serum samples, baseline clinical information and results of diagnostic tests were gathered (see Methods S1). Clinical characteristics were also noted, including whether the patient had a history of surgery or trauma within the week prior to enrollment, history of prior VTE, or current diagnosis of cancer. Comparisons between the two groups were performed using chi-squared binomial testing.

### Clinical Methods

#### D-Dimer measurement

D-Dimer assays were performed by a technician blinded to patient information. Citrated plasma was used for D-dimer ELISA (Dimertest Gold EIA; American Diagnostics, Newport Beach, California), immunoturbidimetric D-dimer assay (BCS; Dade Behring, Marburg, Germany), and D-Di plasma agglutination assay (Diagnostica Stago, Asnieres, France). A cutoff of 90 ng/mL was used as the upper limit of normal for the D-dimer ELISA, based on prior studies demonstrating a sensitivity for thrombosis >95%[13]. For the immunoturbidimetric assay 1.0 mg/L was used as a cutoff. The agglutination assay was interpreted as positive if agglutination occurred in an undiluted specimen.

#### Radiologic Interpretation of VTE

Radiologic studies were performed per clinical standard of care at the hospital. Ultrasound examinations included compression assessment of the deep veins. Prospective investigation of pulmonary embolism diagnosis (PIOPED) criteria were used for lung scan interpretation[14]. Final interpretations were made by the attending radiologist, who was blinded to D-dimer results. All additional radiologic imaging was performed within seven days after the index study, and autopsy results for patients who died within seven days of enrollment were included. VTE was defined as the presence of an acute thrombus by ultrasound, CT scan, angiography, or autopsy. Proximal lower-extremity deep vein thromboses were included in this study when identified at the popliteal level or higher. High probability lung scans were considered positive.

### Proteomic Methods

A brief description of the proteomic methods is provided here, with full details in Methods S1.

#### Direct MALDI-TOF mass spectrometry

Serum samples from patients with and without VTE were analyzed alternatively after random selection of run order of each group. Serum samples were thawed to 4 degrees Celsius, diluted 1∶75 in deionized HPLC-grade water and acidified with 0.1% trifluoroacetic acid. Lactalbumin, which has a molecular weight of 14.2 kDa, was added as an internal standard, or spike protein. The mixture of serum and lactalbumin was co-crystallized in a 1:1 mixture with 3,4-dihydroxycinnamic acid matrix prepared in 50% water, 50% methanol and 0.1% (v/v/v) TFA. Per serum sample, five MALDI-TOF targets were prepared on a gold-coated plate. A total of 4 microliters of serum were used for preparation of the sample and 3.3 nanoliters were ultimately applied to each MALDI target spot. The final concentration of the internal standard protein was 140.8 pM. Per sample, 14 protein expression profiles were obtained for further computational analysis, using a Voyager-DE STR Biospectrometry Workstation MALDI-TOF mass spectrometer (Applied Biosystems, Foster City, California).

#### Computational Analysis

**Figure 1:**
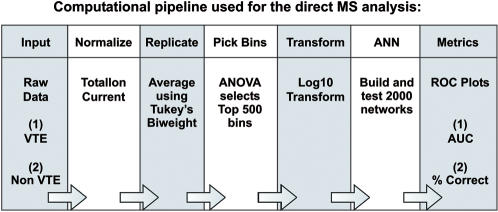
Computational pipeline used for analysis of direct MS data. Successive computational steps were performed using a pipeline to normalize, average and transform mass spectral data, perform ANN analysis and establish performance metrics.

A computational pipeline was constructed to perform successive computational steps, including data pre-conditioning, selection of training and test sets, data reduction, analysis for protein expression differences, selection of best models, and validation **(Figure 1)**. Further methodologic description can be found in Methods S1 (**Figure S1**).

#### (1) Data Pre-conditioning

Each mass spectrum was normalized to its total ion current. The 14 spectra from each serum sample were then averaged, using a Tukey’s biweight schema, yielding one normalized, averaged spectrum per patient (**Figure S2**). Each final spectrum consisted of over 55,000 measurements of the mass-to-charge (m/z) ratios of the ions, most of which are singly charged. Cluster analysis of the averaged, normalized spectra identified 16 spectra with significantly elevated baseline above the average baseline for the entire dataset (data not shown). Since this baseline offset was not correctable using the normalization schema applied, these spectra were excluded from further analysis in the proof of principle experiments presented, yielding 100 patients available for analysis.

#### (2) Partitioning of the Cohort

The study cohort of 100 patients contained 38 patients with VTE. To balance the analyses, 38 patients without VTE were randomly selected for analysis, excluding those spectra with baseline offset. The final study cohort consisted of 76 patients and was randomly divided into three groups: (1) a training set, to formulate the parameters for automated classification, (2) a first test set (test set 1), to select classification parameters that generalize best on new data, and (3) a second test set (test set 2) that was completely withheld for validation testing of the final system. The training set consisted of 38 patients, test set 1 consisted of 20 patients and test set 2 consisted of the remaining 18 patients. Each group contained equal proportions of patients with and without VTE.

#### (3) Mass spectral data reduction

To select the spectral signals representative of relevant protein expression differences, an ANOVA was applied across the mass spectra in the training set. After ranking by significance of the p-value, the 500 most significant bins were selected for further model development.

#### (4) Analysis of protein expression differences

The 500 selected spectral signals were log transformed (base 10), and artificial neural network (ANN) analysis was performed. An ANN was trained on the training set to develop model discrimination parameters. The ANN was designed with a feed forward architecture with a scaled conjugate gradient using MATLAB (The MathWorks, Inc, 2002 v.6.5.018091a Release 13). The trained network contained internal parameters called interconnection weights that served as model parameters that discriminate between the patient groups within the training set.

The ability of the ANN to generalize on new data was then optimized. 2000 ANNs were trained, each with different randomized values for the initial weights. This repetition allowed for greater sampling of the possible sets of model parameters. Each network was tested on test set 1, using the clinically relevant receiver-operator characteristic (ROC) curve, plotting (sensitivity vs. 1-specificity) to assess performance[15]. Area under the curve (AUC) and percent correct were the primary endpoints for analysis. The network with the best performance was selected for further validation testing in test set 2. The AUC based on performance in test set 2 was computed, and sensitivity and specificity were determined.

#### Gel Electrophoresis Analysis of Serum

Gel analyses were performed on four patients with VTE and four age and gender matched controls. Whole serum and serum conditioned for gel electrophoresis were analyzed. Conditioned serum was prepared according to methods recently developed to provide optimal results for gel analysis of serum[16]. Briefly, serum was delipidated and depleted of albumin and IgG (HiTrap protein G HP, Amersham, Piscataway, NJ). Serum proteins were precipitated, resuspended in SDS solubilization buffer (1% SDS, 10 mM HEPES, pH7.4) and quantitated.

#### 1-Dimensional Gel Electrophoresis

1DGE was performed using precast 4–12% NuPAGE gels (Invitrogen, Carlsbad, CA). Serum proteins (2 µl of whole serum or 17 µg of depleted serum) were mixed with lithium dodecyl sulfate sample buffer and heated at 70° C for 10 minutes prior to loading. After electrophoretic separation, proteins were visualized with Coomassie staining (CBB-R250, BioRad Laboratories, Hercules, CA).

#### 2-Dimensional Gel Electrophoresis

2DGE and silver staining of the resulting gels were performed using methods previously published[16] (see Methods S1). 200 µg of protein from the preparation of conditioned serum was analyzed on each gel. Gels were run and stained in pairs, containing one patient with VTE and a paired control without VTE.

#### Computer-assisted gel image analysis

A digital image of each gel was acquired with a PowerLookII scanner at 600 dpi (UMAX Technologies, Inc., Dallas, TX). Protein spots or bands were aligned using Progenesis™ Workstation software (C2005, Nonlinear Dynamic, Newcastle, UK).

#### MS protein identification

After 2DGE, spots were manually excised and digested for MS/MS analysis, using methods described in the Methods S1 section. Data analysis was performed using an NIH-supported version of Mascot (Matrix Science, London, UK).

#### Evaluation of Reproducibility of Methods:

Regarding the MS analysis of serum and computational analysis, MS is a very precise tool for evaluating the mass of proteins, represented as the m/z ratio, on the x-axis of the mass spectrum. The intensity of peaks obtained in mass spectral data (which is displayed on the y-axis of the mass spectrum), however, are prone to variability. Therefore, we used several replicates per patient analyzed. To document the effectiveness of replication in reducing variability, we analyzed a single serum sample from one patient 100 times, using the final method, and calculated a coefficient of variation (CV) across the replicates (CV = 0.10). A CV of 0.15 or less generally indicates highly reproducible data, and thus, we were satisfied that replication is an effective strategy to reduce variability for our analysis of mass spectra derived from serum. In our study, we analyzed 14 replicates per patient. Across the entire spectrum of m/z ratios, the CV with this number of replicates ranged from 0 to 1, with typical values near 0.8. The bins selected for use in the classifier, as described in the section “Mass spectral data reduction,” showed a CV in the range of 0.3 or less. These CVs are in an acceptable range to demonstrate reproducibility of the data used by the classifier to distinguish VTE and non-VTE groups.

For the one- and two-dimensional gel electrophoresis analyses, we applied methods that have been published[16] and demonstrate a high degree of reproducibility of the final gel results. MS-based identification of proteins separated by gel electrophoresis was validated as well, with highly reproducible results from multiple gels[16].

## Results

### Clinical Characteristics

Baseline clinical characteristics were compared between the patient groups. Among the 100 patients eligible for the study and 76 patients ultimately analyzed, there were no differences between patients with and without VTE, with respect to age, gender, history of recent surgery or trauma, or presence of advanced malignancy (**Table 1**). Patients with VTE did more often have a history of prior VTE (p = 0.0003). CRP and fibrinogen levels were not significantly different between the two groups (Table 1).

**Table 1:** Baseline characteristics of the patient cohort*.

(a) 100 patients eligible for analysis				
	All (N = 100)	VTE (N = 38)	No VTE (N = 62)	
Age-years	55.92±16.59	54.92±18.94	56.53±15.11	p = 0.6398
Male-no. (%)	41 (41.0)	14 (36.84)	27 (43.55)	p = 0.5081
Post-operative-no. (%)	28 (28.0)	11 (28.95)	17 (27.42)	p = 0.8688
Cancer-no. (%)	33 (33.0)	16 (42.11)	17 (27.42)	p = 0.1295
Prior VTE-no. (%)	17 (17.0)	13 (34.21)	4 (6.45)	**p = 0.0003**
Hypertension-no. (%)	10 (10.0)	4 (10.53)	6 (9.68)	p = 0.8921
Diabetes-no. (%)	5 (5.0)	2 (5.26)	3 (4.84)	p = 0.9256
Renal dysfunction-no. (%)	3 (3.0)	0 (0)	3 (4.84)	p = 0.1719
Pulmonary disease-no. (%)	4 (4.0)	0 (0)	4 (6.45)	p = 0.1122
Coronary artery disease-no. (%)	3 (3.0)	1 (2.63)	2 (3.23)	p = 0.8674
Congestive heart failure-no. (%)	4 (4.0)	1 (2.63)	3 (4.84)	p = 0.5890
Hepatic dysfunction-no. (%)	3 (3.0)	0 (0)	3 (4.84)	p = 0.1719
HIV-no. (%)	1 (1.0)	0 (0)	1 (1.61)	p = 0.4365
Plasma CRP-µg/ml	5.90±7.04	5.06±5.05	6.41±8.02	p = 0.3566

(b) 76 patients selected for analysis				
	All (N = 76)	VTE (N = 38)	No VTE (N = 38)	
Age-years	55.57±16.99	54.921±18.94	56.21±15.02	P = 0.7432
Male-no. (%)	32 (42.11)	14 (36.84)	18 (47.37)	P = 0.3527
Post-operative-no. (%)	22 (28.95)	11 (28.95)	11 (28.95)	p = 1.0
Cancer-no. (%)	25 (32.89)	16 (42.11)	9 (23.68)	p = 0.0874
Prior VTE-no. (%)	15 (19.74)	13 (34.21)	2 (5.26)	**p = 0.0015**
Hypertension-no. (%)	8 (10.53)	4 (10.53)	4 (10.53)	p = 1.0
Diabetes-no. (%)	3 (3.95)	2 (5.26)	1 (2.63)	p = 0.5621
Renal dysfunction-no. (%)	2 (2.63)	0 (0)	2 (5.26)	p = 0.1600
Pulmonary disease-no. (%)	2 (2.63)	0 (0)	2 (5.26)	p = 0.1600
Coronary artery disease-no. (%)	2 (2.63)	1 (2.63)	1 (2.63)	p = 1.0
Congestive heart failure-no. (%)	3 (3.95)	2 (5.26)	1 (2.63)	p = 0.5621
Hepatic dysfunction-no. (%)	2 (2.63)	0 (0)	2 (5.26)	p = 0.1600
HIV-no. (%)	1 (1.32)	1 (2.63)	0 (0)	p = 0.3238
Plasma CRP-µg/ml	5.70±7.37	5.06±5.05	6.33±9.16	p = 0.4561
Fibrinogen-mg/ml	470.64±212.71	460.87±195.88	480.15±230.09	p = 0.6930

* Plus-minus values are means±SD

Diagnostic radiographic imaging modalities used to assess whether VTE was present included ultrasound imaging of the extremities, V/Q scans, CT scans, venography and pulmonary angiography. The overall proportion of imaging modalities used was not significantly different between the patient groups, comparing the use of each individual modality between the groups. Among patients diagnosed with VTE, 13 patients (34.2%) had DVT only, 19 (50%) had PE only and 6 (15.8%) were diagnosed with both DVT and PE.

### Direct mass spectrometry analysis of serum

**Figure 2:**
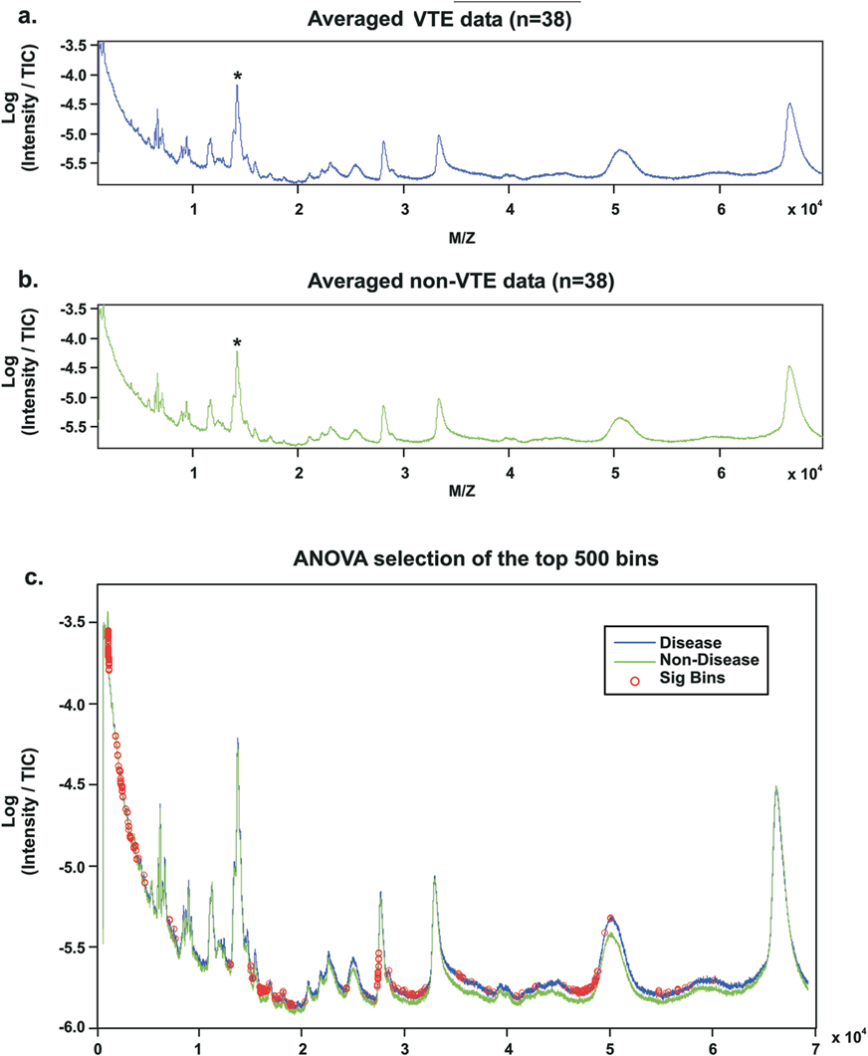
Direct MS of serum. Average spectra for patients with VTE (a) and without VTE (b) show differences in overall protein expression, with the internal spike protein denoted by an asterisk. The 500 signals selected by the ANOVA analysis are denoted by red circles, representing locations on the mass spectrum with differential protein expression between the two groups of patients (c).

Individual serum analysis yielded a reproducible spectrum in which the spike protein was visualized in 100% of samples analyzed (**Figure 2a, 2b**). Expression of the internal spiked-in control protein showed equivalent intensity across all samples, with a CV of 0.034. No difference in expression was noted of the spiked-in protein in the ANOVA between patients with and without VTE (p = 0.53). Correlation (r^2^) of all m/z ratios for the 14 spectra acquired per patient was 0.9601 (standard deviation 0.0170). The average correlation between patients was 0.9593 (standard deviation 0.044). These measures of inter- and intra-individual variability are consistent with other, similar proteomic methodologies**[17], [18]**.

To explore overall changes in protein expression, an average spectrum was computed from all patients with VTE and another from patients without VTE. The ANOVA applied across all mass spectral data, comparing VTE and NoVTE spectra, yielded a p-value for each point on the x-axis of the mass spectra. Rank ordering the p-values, the 500 smallest p-values all were p<0.033, without multiple testing corrections (**Figure 2c**).

### ANN-based classification of protein expression profiles

**Figure 3:**
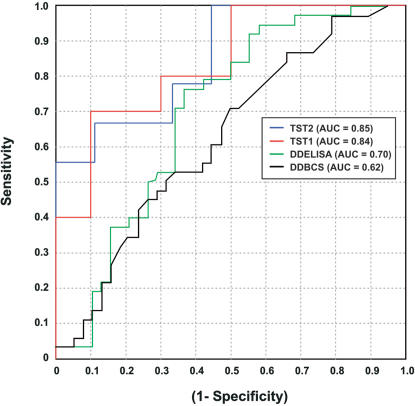
ROC characteristics for the direct MS method in the independent test set compared to standard D-dimer tests. The optimized model shows comparable ROC curves in test set 1 (TST1) and test set 2 (TST2). D-Dimer by ELISA and immunoturbimetric assay results are plotted for all 76 patients in the cohort for comparison.

The ANN-based class prediction model developed on the training set and optimized in test set 1 ultimately classified 78% of patients correctly in test set 2. In this, blinded independent validation group, 77% of patients with VTE were correctly classified and 89% of patients without VTE were correctly classified. The specificity of the model exceeded the specificity of any D-dimer method in tested in both the validation group as well as the entire cohort, tested by latex agglutination, ELISA, and immunoturbimetric methods which showed sensitivity/specificity of 63.2%/60.5%, 97.4%/21.1%, 97.4%/15.8%, respectively (**Table 2**). In the validation group, an AUC of 0.85 was determined by plotting the ROC of sensitivity vs. (1-specificity) (**Figure 3**). The AUC for blood D-dimer levels assessed by ELISA and immunoturbimetric methods were 0.70 and 0.62 respectively. Examining the ROC curves for the two D-dimer tests with continuous values reported by the clinical laboratory on all 76 patients studied, the ANN-based prediction model performed best. Using a resampling method to generate confidence intervals and a parametric t-test to compare AUCs in test set 2, the improved ANN performance is significant (p<0.001).

**Table 2:** Performance of the direct MS method of assessing protein markers of VTE compared to d-dimer.

Overall cohort: 100 patients		
	Sensitivity	Specificity
Latex agglutination d-dimer	63.20%	58.10%
ELISA d-dimer	97.30%	19.40%
Immunoturbimetric d-dimer	97.30%	16.10%
**Overall cohort: 76 patients**		
	Sensitivity	Specificity
Latex agglutination d-dimer	63.20%	60.50%
ELISA d-dimer	97.40%	21.10%
Immunoturbimetric d-dimer	97.40%	15.80%
**Validation Group: 18 patients**		
	Sensitivity	Specificity
Latex agglutination d-dimer	55.60%	77.80%
ELISA d-dimer	100%	33.30%
Immunoturbimetric d-dimer	100%	22.20%
Direct MS method	67.70%	88.90%

Sensitivity and specificity of the direct MS method is compared to the results of standard clinical d-dimer assays performed in the cohort.

To examine possible confounding effects of inflammation, introduced through prior VTE or concurrent cancer diagnosis, misclassification rates were tabulated. Chi-squared statistics (df = 3) demonstrated no significant relationship between CRP and classification errors (p = 0.72 in all patients, n = 78; p = 0.27 in test set 2, n = 18).

### Gel electrophoresis of serum proteins

1DGE and 2DGE were next undertaken on a subset of the cohort, to determine whether serum protein expression differences that were detected and used by the ANN-based classification method of direct MS data could be validated by established proteomic techniques used to characterize protein expression.

**Figure 4:**
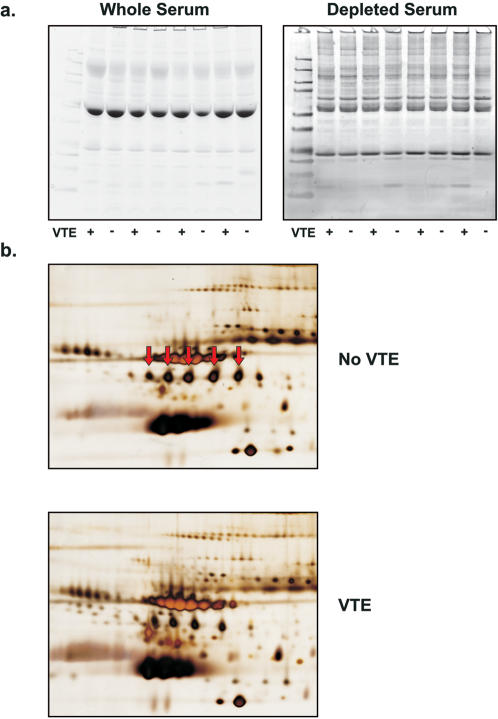
Gel electrophoresis of serum from patients with and without VTE. 1-DGE of whole serum and depleted, conditioned serum was run on 4 VTE and 4 NonVTE patients (a). 2-DGE was performed on serum from patients with VTE and without VTE, with arrows pointing to a series of haptoglobin proteins with differential expression between VTE and NonVTE patients, displayed in two representative gels (b).

1DGE performed on whole serum showed very few protein bands other than albumin and no discernable differences between serum samples from patients with and without VTE (**Figure 4a**). 1DGE of conditioned serum, that had been delipidated and depleted of high abundance proteins, showed many distinct bands of varying intensity. However, no clear patterns between the patient groups were discerned with visual inspection or computer-assisted analysis (**Figure 4a**). 2DGE demonstrated differences in serum protein expression between patients with and without VTE (**Figure 4b**). Several spots were excised from the gel and successfully identified using MS/MS for sequence-based protein identification (**Supplemental Table S1**). Proteins identified include haptoglobin, platelet coagulation factor XI, complement 9, plasma kallikrein B1 precursor, immunoglobulin alpha-1 heavy chain constant region, proapolipoprotein and alpha-1B glycoproteins (**Figure S3**). Proteins known to be of relatively high abundance in human serum were easily detected by visual inspection of serial gels. Differential expression was noted of proteins such as haptoglobin, alpha-1B glycoproteins, and others, validating that protein expression differences are present in serum of patients with and without VTE.

## Discussion

Clinical biomarkers used to diagnose cardiovascular disease typically consist of single protein markers. However, since multiple proteins interact in processes such as thrombosis, it is reasonable to hypothesize that a panel of multiple protein markers may more accurately detect a disease. We hypothesized that an intravascular thrombus would result in protein expression differences that could be detected using direct MS. After establishing a reproducible and relatively simple direct MS method to analyze serum, we successfully applied advanced computational methods to develop a discrimination model. Final validation of the model in an independent, withheld subset of the data demonstrated enhanced diagnostic performance compared to routinely available D-dimer assays. Gel-based analyses validated that protein expression differences between the patient groups are present among proteins with relatively high abundance in serum.

We used a direct MS method to analyze serum in this study, using MALDI-TOF MS. Other clinical proteomic approaches using direct MS have typically employed a technology known as Surface Enhanced Laser Desorption Ionization (SELDI) to enrich for subsets of proteins on the MS sample target surface[10], [19]. The advantage of this approach is that the protein mixture is simplified, in that only those proteins with a specific biochemical property adhere to the given SELDI surface, providing stronger MS signals from the enriched set of captured proteins. However, this process may alter relative protein abundance, potentially diminishing the ability to detect meaningful differences in relative protein expression of those proteins with sufficient abundance to detect without such pre-enrichment steps. Additionally, technical issues such as inter- and intra-chip variation can be an issue with SELDI methodologies. Therefore, the relative utility of our method and established SELDI approaches is unknown. We chose to apply MALDI-TOF MS on serum processed minimally, aiming to preserve relative protein expression levels as close to the original serum sample as possible. Further, we go on to validate protein expression differences using gel-based proteomic methods.

The approach to data analysis in this study deserves specific consideration in light of potential pitfalls of direct MS methodologies. Given that mass spectra are prone to variability in ionization efficiency, we applied a standardized method of analyzing serum directly with MALDI-TOF MS, using averaging of technical replicates and normalization to a measure of total ionization efficiency by the laser. Technical replication and normalization of data are well-validated approaches to deal with variability in other genomic methodologies, such as microarray transcript expression profiling[20]. Consistency of the internal spike protein across samples demonstrates that variability of MS data can be successfully managed using these methods.

Machine learning methods applied to direct MS data carry risks of confounding, bias, and overfitting[21]. Confounding and bias were avoided by using available serum samples without selection for this study, from a single enrollment center in which all samples were handled identically, to minimize the influence of pre-analytic factors. Sample analysis was conducted in a blinded manner. Overfitting is a problem commonly encountered in genomics research, in which a large number of potential predictors are used to discriminate between patient outcome groups[22]. This is clearly a risk in the analysis of direct MS data[23]–[25]. To minimize this risk, we applied an exceptionally stringent approach that divides the study cohort into a training set and two test sets. Despite the resulting diminished sample size in each group, we demonstrate enhanced diagnostic performance of the direct MS method using the well-validated receiver operator curve characteristics. Thus, we are able to directly compare sensitivity and specificity of the proteomic expression profiles to currently available single marker tests for D-dimer.

Limitations of this study include the determination of VTE outcome as that made in the routine clinical setting. The gold standard for pulmonary embolus is pulmonary angiography, and for deep venous thrombosis, it is venography[26]–[29]. An observational approach was used in this study, given the exploratory nature of the analyses performed here. Testing was conducted with a small sample size, and further validation in a larger study will be needed to verify these results. In addition, MS patterns can be studied in two additional groups: patients with a history of VTE but not an acute VTE (examining the proteomic profile of chronic VTE in the absence of an acute event); and patients with a chronic inflammatory condition, in the absence of VTE. These two additional groups would address the MS pattern chronic inflammation or chronic VTE and overlap with acute VTE. These studies will be conducted in future work.

To address whether the detected MS signals used by the model to categorize patients with and without VTE do in fact reflect protein expression differences, we present focused data from 2DGE corroborating differential protein expression identified between the patient groups. This suggests that the direct MS method is capable of detecting differences in protein expression in whole serum of those proteins of sufficient abundance to be detected by 2DGE performed on depleted serum. Further, our findings suggest that direct MS, a relatively simply analytic method using very little serum, may potentially be useful for guiding subsequent proteomic analyses aimed to identify specific protein markers of disease. Specific validation of protein markers identified in this study requires further focused examination of these proteins.

We performed this pilot investigation in a population of hospitalized patients. These patients had other potential confounders including recent trauma, surgery and cancer, which are known to alter predisposition to thrombosis and may alter coagulation protein expression patterns in the absence of an intravascular thrombus. Additionally, prior VTE was found to be more common among patients with acute VTE, consistent with other studies but also possibly acting as an additional confounder. Reliable blood based assays would be of value in this population, where currently available markers of VTE suffer from low specificity[30]. We chose this more diagnostically challenging group of patients for the proof of principle experiment presented here, to determine if this method could detect protein expression differences against a biologically “noisy” background. Since the direct MS method was able to detect meaningful changes in this population, we hypothesize that the method will be robust to patient-to-patient variation and other sources of variability that would be commonly encountered in clinical application.

Given that the direct MS method described represents a noninvasive test that can be performed rapidly, inexpensively and in an automated fashion, requiring only 10 nanoliters of serum, this technology holds promise for clinical application. As FDA-sponsored studies are ongoing to evaluate clinical utility of protein expression patterns using SELDI-TOF MS for the diagnosis of diseases such as cancer, direct MALDI-TOF of serum may be a complementary approach deserving of consideration for applications such as VTE[31]. Importantly, validation of novel biomarkers requires rigorous testing in large cohorts to determine the potential clinical utility[15]. In the case of VTE, testing diagnostic utility in conjunction with clinical risk stratification and radiographic modalities will be essential[32]–[34]. While currently available diagnostic protocols using the D-dimer assay provides a high negative predictive value, the proteomic markers we identified appear to add specificity to currently available blood assays. In the future, a combination of methods may provide optimal sensitivity and specificity, including ultrasound compression, D-dimer testing and possibly a MS test to help clarify situations in which the diagnosis of DVT and PE could not be made by the first two tests. In addition, with further research, we may be able to develop a potential model using all modalities, combining new markers identified through proteomic methods such as ours and future efforts to further refine these, in combination with currently available clinical methods such as D-dimer testing and compression ultrasound. Our analysis provides the basis for proposing a potential model using proteomic modalities in combination with currently available clinical methods, such as D-dimer testing, compression ultrasonography and radiographic testing for VTE. We envision that future practical application will likely require further independent validation of our findings and further proteomic analysis to identify the specific markers underlying the mass spectral signals detected in our work.

## Supporting Information

Methods S1 Supplemental Methods Section
(0.25 MB DOC)

Supplemental Table S1 (0.03 MB DOC)

Figure S1 Computational pipeline used for analysis of direct MS data. Successive computational steps were performed using a pipeline to normalize, average and transform mass spectral data, perform ANN analysis and establish performance metrics.
(11.66 MB TIF)

Figure S2 Averaging of mass spectra per patient. For 4 different patients studied, the 14 raw mass spectra are shown, with the final averaged spectrum below each set of raw mass spectra.
(11.65 MB TIF)

Figure S3 MS/MS data for haptoglobin. The raw chromatogram is shown (a, upper panel) along with the chromatogram for the peptide YVMLPVADQDQCIR with observed mass of 854.41 Da (a, lower panel). MS of the peptide is shown (b), followed by the raw MS/MS data (c).
(11.66 MB TIF)
