# Supplementary material for: Detection of Venous Thromboembolism by Proteomic Serum Biomarkers
Source: PLoS One. 2007 Jun 20;2(6):e544. doi: 10.1371/journal.pone.0000544 (PMC1891085; doi:10.1371/journal.pone.0000544)
Supplement: Methods S1 — Supplemental Methods Section (0.25 MB DOC) [file pone.0000544.s001.doc]

**Supplemental Methods Section (Methods S1)**

**PROTEOMIC METHODS**

**Serum sample collection and handling**

Whole blood was collected into standard serum separator tubes and clotted for approximately 2 hours at room temperature prior to centrifugation. The samples were thawed only once after initial freeze at -80 degrees Celsius prior to direct MS analysis.

**Direct MALDI-TOF mass spectrometry**

Serum samples were thawed to 4 degrees Celsius, diluted 1:75 in deionized HPLC-grade water and acidified with 0.1% trifluoroacetic acid. Lactalbumin, which has a molecular weight of 14.2 kDa, was added as a spike protein. Various proteins were tested for suitability as an internal standard, including bee mellitin, bromelain, and others. Lactalbumin yielded the most consistent signal at the MS acquisition settings used and was therefore chosen as the internal standard for this study. The mixture of serum and lactalbumin was co-crystallized in a 1:1 mixture with 3,4-dihydroxycinnamic acid matrix prepared in 50% water, 50% methanol and 0.1% TFA. Per serum sample, five MALDI-TOF targets were prepared on a gold-coated plate. A total of 4 microliters of serum were used for preparation of the sample and 3.3 nanoliters were ultimately applied to each MALDI target spot. Protein expression profiles were obtained using a Voyager-DE STR Biospectrometry Workstation MALDI-TOF mass spectrometer (Applied Biosystems, Foster City, California).

Variables influencing sample processing, including concentration and acidity of the final sample, method for spotting on the MALDI plate and strength of laser ionization were optimized in a set of variance experiments. The final method yielded a CV of 0.10 in the analysis of 100 replicates. This standardized method of serum processing and MS data acquisition was applied to all serum samples. Data were acquired in linear mode using a relatively low and constant laser setting of 1948. Each mass spectrum was obtained using a pre-specified acquisition pattern from each spot, and 100 shots were averaged by the mass spectrometer to yield each raw mass spectrum. Per sample, 14 spectra were acquired for final analysis. Serum samples from patients with and without VTE were analyzed alternatively after random selection of run order of each group.

**Computational Analysis**

The computational analysis pipeline performed successive steps as outlined in **Figure S1**.


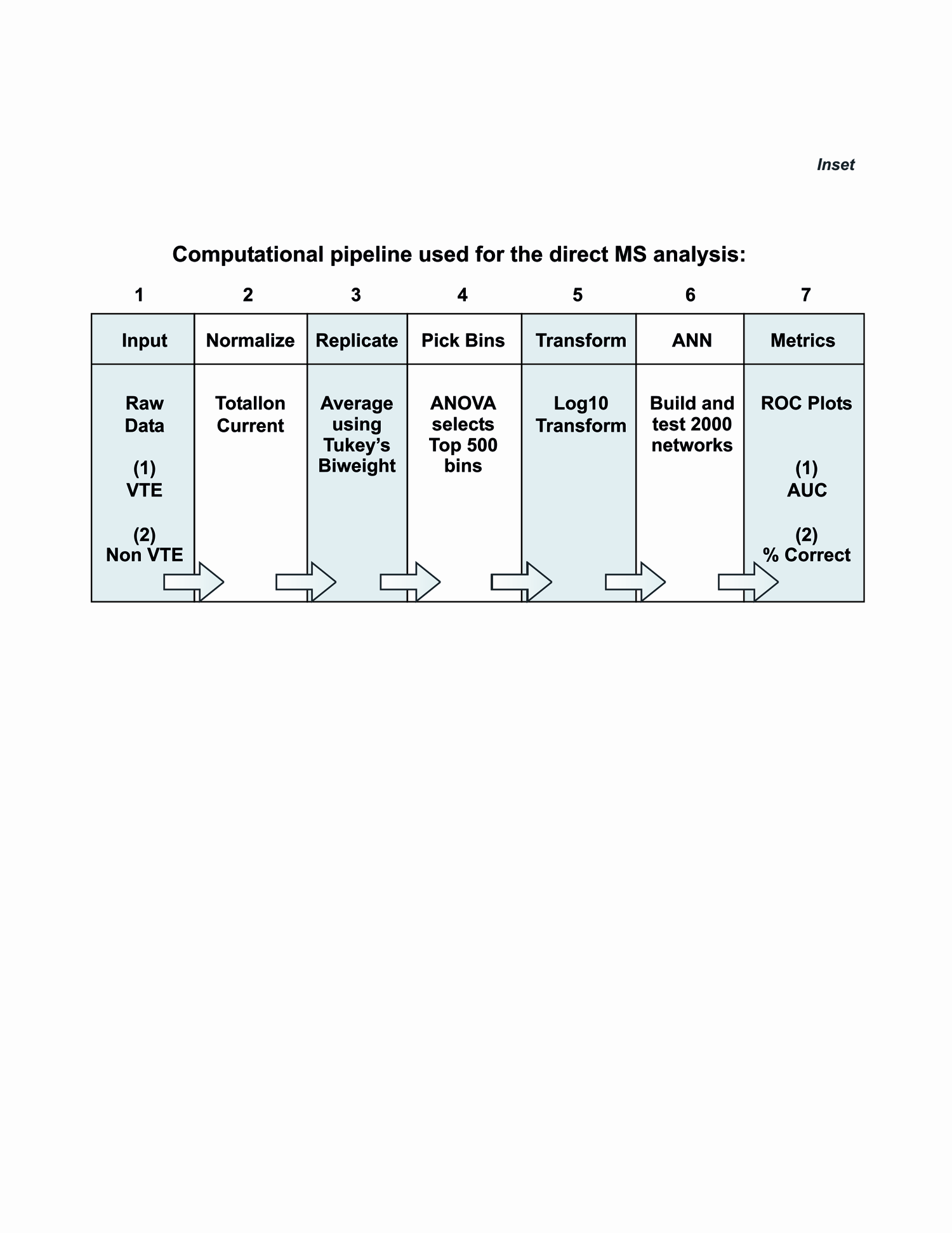


1. Input: Input to the computational pipeline included the raw mass spectra obtained from each patient.

2. Normalize: Normalization was performed to the total ion current of each mass spectrum. Normalization was performed using two methods: normalizing to the peak intensity of the lactalbumin peak at approximately 14.2 kDa and the total ion current. CV was calculated on a bin by bin basis across the mass spectra for each patient and then averaged across all mass spectra. Normalization to the total ion current (CV =1.0616) was chosen over normalization to the lactalbumin signal (CV= 1.4516). Adjustment of the data on the x-axis was not performed since the x-axis locations of expected matrix signals and peak intensity of the lactalbumin spike were uniform between samples, consistent with our expectations, given that the MALDI acquisition settings included automatic calibration of each raw mass spectrum to expected matrix signals for 3,4-dihydroxycinnamic acid prior to output from the mass spectrometer.

3. Replicates: The normalized spectra were then averaged per patient, yielding one final averaged mass spectrum per patient. A Tukey’s biweight schema was used for averaging since outliers are handled better with this method (**Figure S2**).

4. Bin selection: An ANOVA was applied across the mass spectra between patients with and without VTE in the training set, on a bin by bin basis, meaning that a p-value was determined by this univariate test per data point on the mass spectrum. The p-values were then ranked, and the bins corresponding to the smallest 500 uncorrected p-values were selected for further analysis with the artificial neural network (ANN).

5. Transformation: Prior to ANN analysis, the data was log transformed (base 10) in order to more readily detect signals close to the level of noise.

6. ANN: 2000 neural networks were built with the selected 500 mass spectral data points, using the training set.

7. Performance metrics: The networks were each tested on test set 1, with receiver-operator characteristics (ROC), plotted as sensitivity versus (1-specificity) yielding an area under the curve (AUC). The model that yielded the highest AUC was selected for final validation testing in the test set, assessed again by ROC plot characteristics and percent correct classification.

**2-Dimensional Gel Electrophoresis**

2DGE was performed using methods previously published [1]. 200 ug of protein from conditioned serum precipitated and resuspended in rehydration buffer (8 M urea, 2 M thiourea, 5% (w/v) DTT, 1% (v/v) Hydroxy-Ethyl-Disulfide (HED), 0.2% (v/v) carrier ampholytes (pH 4-7), and a trace amount of bromophenol blue. Isoelectric focusing was first performed. The resuspended proteins were actively rehydrated at 50 Volts (V) for 16 hours on 24 cm pH4-7 IPG strips (Amersham Biosciences, Uppsala, Sweden) using a Protein IEF cell (BioRad, Hercules, CA). After rehydration, the following voltages were applied to the IPG strips: 150V for 1.5 hours, 300V for 2 hours, 600V for 1 hour, a 2 hour linear ramp up to 10,000V, and finally 10,000V for 4.5 hours. All strips were reduced and alkylated for 20 minutes each with 130 mM dithiothreitol then 135 mM iodoacetamide in 50 mM Bis-Tris (pH 6.4), 6M urea, 30% (w/v) glycerol, 2% (w/v) LDS.

Equilibrated strips were subjected to electrophoresis on hand-cast 10% polyacrylamide gels with an ionicity of 375 mM Bis-Tris (pH 6.4) in the resolving gel. A stacking gel of 5% acrylamide and 125 mM Bis-Tris (pH 6.4) with a trace amount of bromophenol blue was used to embed the strip in the gel. The running buffer contained 50 mM MES, 50mM Tris-base, 3.5 mM SDS and 1mM EDTA. Electrophoresis was performed in a PROTEAN plus Dodeca cell horizontal system (BioRad) with 50V at 0.5 hour followed by 75V for 12 hours. Gels were stopped when the bromophenol blue dye front reached the bottom of the gel.

Silver staining was performed using previously described methods[2]. Briefly, gels were fixed in 50% methanol and 5% acetic acid for 1 hour and washed with Milli-Q water. Gels were sensitized with 0.02% (w/v) sodium thiosulfate for 1 minute, washed, and placed in 0.1% silver nitrate for 20 minutes. Gels were than washed with water and developed with 2% (w/v) sodium carbonate containing 0.015% (v/v) formaldehyde. Development was stopped with 5% acetic acid, and gels were stored in 1% acetic acid. Gels were run and stained in pairs, containing one patient with VTE and a paired control without VTE.

**MS protein identification**

After 2DGE, spots were manually excised and digested for MS/MS analysis. The spots were destained in 15 mM potassium ferricyanide/50 mM sodium thiosulfate, reduced in 10 mM DTT/100 mM ammonium bicarbonate for 30 minutes and alkylated in 55 mM iodoacetamide/100 mM ammonium bicarbonate for 20 minutes. The gel pieces were washed in 100 mM ammonium bicarbonate and dehydrated in ACN. The peptides were digested with 6 ng/mL trypsin in 50 mM ammonium bicarbonate for 5 hours at 37° C. The peptides were extracted using 1% formic acid/2% ACN, followed by two extractions with 50% ACN. The extracts were pooled and dried under vacuum.

Lyophilized digestion samples reconstituted in 12 ul of 0.1% formic acid were injected into an Agilent HP1100 CapLC upfront from a Waters Micromass QTOF Ultima Global spectrometer (Manchester UK).The column was a Grace Vydec C-18 reversed phase (10cmX 150 uM, The Nest Group, Southboro, MA) and the solvent eluted at 1.0 uL/minute. Data-dependent analysis was performed on the top three most abundant multiply charged precursor ions detected in each MS scan (m/z 300-1990; 1.0 sec/scan). MS/MS scans (m/z 50-1990) were switched back to MS according to (1) TIC threshold rising above 3000 counts, or (2) elapsed time of no more than 6 sec (2.0 sec per MS/MS scan). TOF MS survey scan was 350–1200 atomic mass units for 0.5 second, followed by first, second and third product MS/MS (65–1200 amu for 1.5 seconds). Data analysis was performed using an NIH-supported version of Mascot (Matrix Science, London, UK) (**Figure S3**).

**Mascot search for protein identification**: The raw chromatogram for each MS/MS scan was examined, with total ion current (TIC) ranging from 3.76e4 to 1.36e5, with higher TIC corresponding with high confidence protein matches. Error tolerant searches were not performed. Mowse scores greater than 50 were accepted.

Supplemental Methods References:

1. Fu Q, Garnham CP, Elliott ST, Bovenkamp DE, Van Eyk JE (2005) A robust, streamlined, and reproducible method for proteomic analysis of serum by delipidation, albumin and IgG depletion, and two-dimensional gel electrophoresis. Proteomics.

2. Shevchenko A, Wilm M, Vorm O, Mann M (1996) Mass spectrometric sequencing of proteins silver-stained polyacrylamide gels. Anal Chem 68: 850-858.

**Figure Legends**

**Figure S1**

**Computational pipeline used for analysis of direct MS data.** Successive computational steps were performed using a pipeline to normalize, average and transform mass spectral data, perform ANN analysis and establish performance metrics.

**Figure S2**

**Averaging of mass spectra per patient**. For 4 different patients studied, the 14 raw mass spectra are shown, with the final averaged spectrum below each set of raw mass spectra.

**Figure S3**

**MS/MS data for haptoglobin**. The raw chromatogram is shown (a, upper panel) along with the chromatogram for the peptide YVMLPVADQDQCIR with observed mass of 854.41 Da (a, lower panel). MS of the peptide is shown (b), followed by the raw MS/MS data (c).

**References:**

1. Fu Q, Garnham CP, Elliott ST, Bovenkamp DE, Van Eyk JE (2005) A robust, streamlined, and reproducible method for proteomic analysis of serum by delipidation, albumin and IgG depletion, and two-dimensional gel electrophoresis. Proteomics.

2. Shevchenko A, Wilm M, Vorm O, Mann M (1996) Mass spectrometric sequencing of proteins silver-stained polyacrylamide gels. Anal Chem 68: 850-858.
