# Supplementary material for: Detection of Venous Thromboembolism by Proteomic Serum Biomarkers
Source: PLoS One. 2007 Jun 20;2(6):e544. doi: 10.1371/journal.pone.0000544 (PMC1891085; doi:10.1371/journal.pone.0000544)
Supplement: Supplemental Table S1 — (0.03 MB DOC) [file pone.0000544.s002.doc]

**Supplemental Table 1**

* Highest Mowse score and corresponding peptides are reported.

† 1=multiple gel spots contained this protein; 2=no isoform information; 3=cannot determine if this is a pre-protein or mature form; 4=additional peptides from this protein were identified in other spots. **References:**

1. Fu Q, Garnham CP, Elliott ST, Bovenkamp DE, Van Eyk JE (2005) A robust, streamlined, and reproducible method for proteomic analysis of serum by delipidation, albumin and IgG depletion, and two-dimensional gel electrophoresis. Proteomics.

2. Shevchenko A, Wilm M, Vorm O, Mann M (1996) Mass spectrometric sequencing of proteins silver-stained polyacrylamide gels. Anal Chem 68: 850-858.
