# Supplementary figures and images for: Detection of Venous Thromboembolism by Proteomic Serum Biomarkers
Source: PLoS One. 2007 Jun 20;2(6):e544. doi: 10.1371/journal.pone.0000544 (PMC1891085; doi:10.1371/journal.pone.0000544)

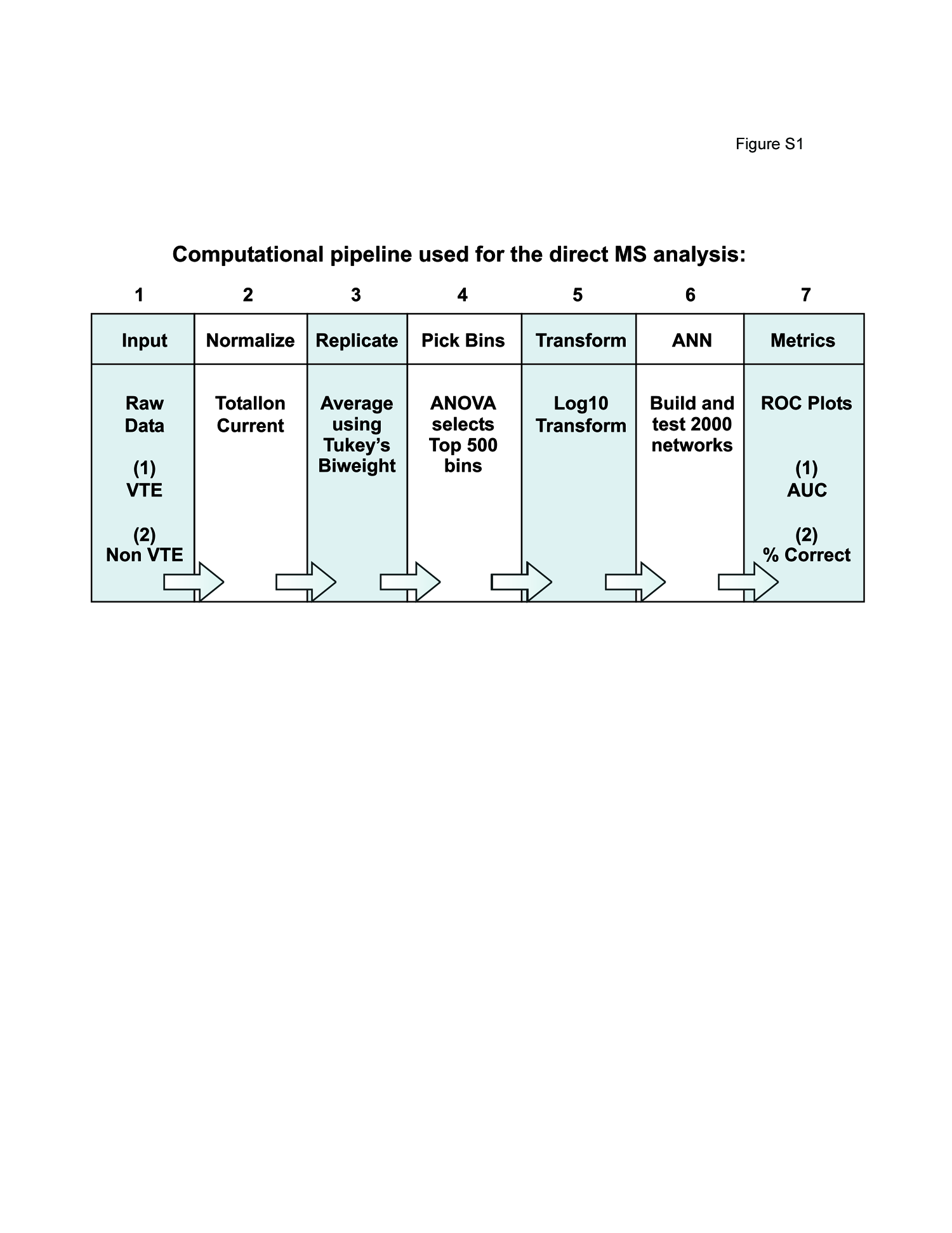

Supplement: Figure S1 — Computational pipeline used for analysis of direct MS data. Successive computational steps were performed using a pipeline to normalize, average and transform mass spectral data, perform ANN analysis and establish performance metrics. (11.66 MB TIF) [file pone.0000544.s003.tif]

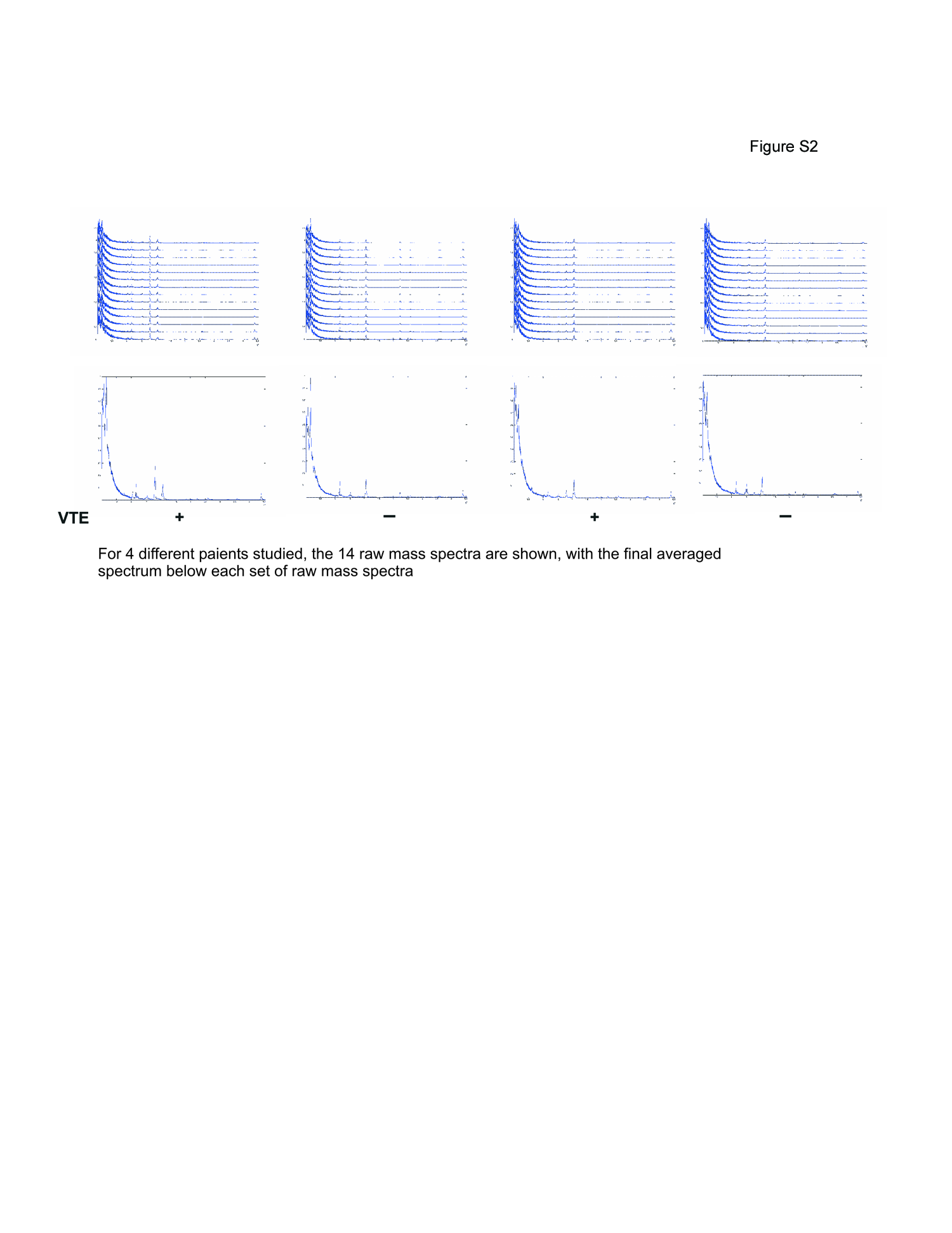

Supplement: Figure S2 — Averaging of mass spectra per patient. For 4 different patients studied, the 14 raw mass spectra are shown, with the final averaged spectrum below each set of raw mass spectra. (11.65 MB TIF) [file pone.0000544.s004.tif]

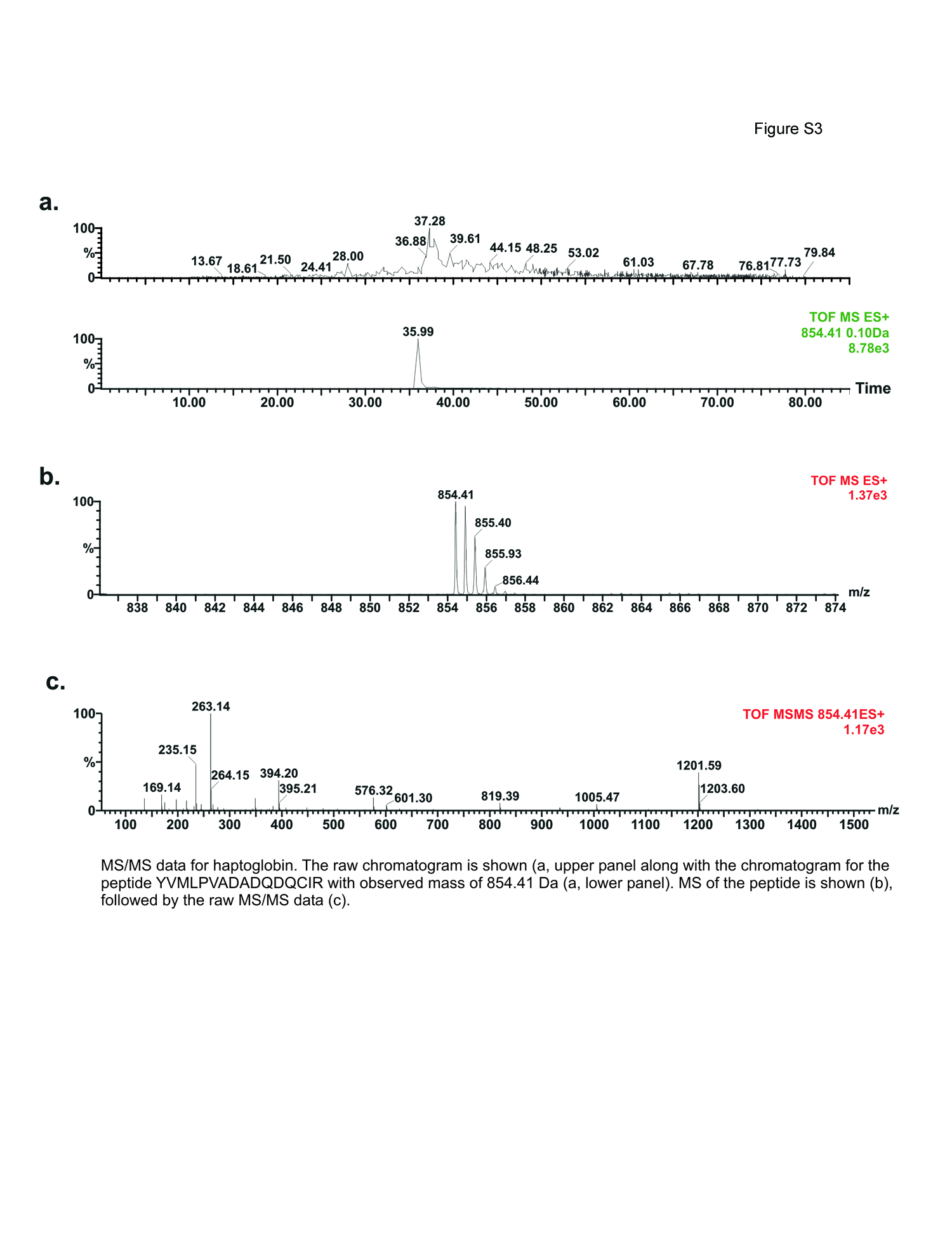

Supplement: Figure S3 — MS/MS data for haptoglobin. The raw chromatogram is shown (a, upper panel) along with the chromatogram for the peptide YVMLPVADQDQCIR with observed mass of 854.41 Da (a, lower panel). MS of the peptide is shown (b), followed by the raw MS/MS data (c). (11.66 MB TIF) [file pone.0000544.s005.tif]
